# Targeting lncRNAs of colorectal cancers with natural products

**DOI:** 10.3389/fphar.2022.1050032

**Published:** 2023-01-09

**Authors:** Woo Jung Sung, Jaewoo Hong

**Affiliations:** ^1^ Department of Pathology, Daegu Catholic University School of Medicine, Daegu, South Korea; ^2^ Department of Physiology, Daegu Catholic University School of Medicine, Daegu, South Korea

**Keywords:** ncRNA, natural products, lysosome, solid tumor, colorectal cancer, lncRNA

## Abstract

Non-coding RNA (ncRNA) is one of the functional classes of RNA that has a regulatory role in various cellular processes, such as modulation of disease onset, progression, and prognosis. ncRNAs, such as microRNAs (miRNAs), long non-coding RNAs (lncRNAs), and circular RNAs (circRNAs), have been actively studied in recent years. The change in ncRNA levels is being actively studied in numerous human diseases, especially auto-immune disorders and cancers; however, targeting and regulating ncRNA with natural products to cure cancer has not been fully established. Recently many groups reported the relationship between ncRNA and natural products showing promising effects to serve as additional therapeutic approaches to cure cancers. This mini-review summarizes the aspects of lncRNAs related to cancer biology focusing on colorectal cancers that natural products can target.

## 1 Introduction

Colorectal cancer (CRC) is the third most common cancer and the fourth most cause of cancer deaths globally (Siddiqui et al., 2019; Hassen et al., 2022). CRC onset has a higher tendency in developed countries than in developing countries (Hassen et al., 2022). Several etiological factors may affect the development of CRC, such as environmental, genetic, and epigenetic factorss (Anupriya et al., 2022). Usually, CRC is developed gradually over 1–2 decades (Siddiqui et al., 2019). The most common initiation of CRC is from adenomatous polyps of colorectal glandular epithelial cells. Malignant CRC begins when adenomatous polyps have mutations in the *Adenomatous polyposis coli* gene, tumor suppressor genes, and/or oncogenes (El Zoghbi and Cummings, 2016). The mortality of CRC increases significantly after metastasis and invasion initiation to other organs and tissues (Dowli et al., 2023). So, elucidating molecular mechanisms of the development and progression of CRC and searching for new markers and therapeutic strategies are essential in both basic and clinical sciences (Yu et al., 2022; Zheng et al., 2022). Recent findings revealed that epigenetic alterations are more frequent than genetic alterations in CRC (Okugawa et al., 2015). Currently, many groups focus on epigenetic studies on CRC to discover new biomarkers for diagnosis and develop new therapies (Ullah et al., 2022). The application of natural products such as phytochemicals with anticancer effects can be considered as one of the approaches to target lncRNAs to treat CRC, which may increase the sensitivity of CRC cells additionally to the prevailing therapies. This review investigates the effect of various phytochemicals on lncRNAs of CRC and evaluates their capacity to treat or prevent CRC.

## 2 LncRNAs

Non-protein coding RNAs with transcripts 200 bp or longer are called lncRNA (Costa et al., 2022; Pagani et al., 2022; Razlansari et al., 2022), which were believed to be byproducts of RNA polymerase II transcription without specific biological actions (Goodrich and Kugel, 2006; Wagner et al., 2013; Nojima and Proudfoot, 2022). Currently, lncRNA does not have a standardized classification. However, lncRNAs are classified by their location, such as cytoplasmic, nuclear, and cytoplasmic nuclear lncRNAs (Kerachian and Azghandi, 2022) and they have different regulatory functions where they are located (Ghafouri-Fard et al., 2022). Mainly, cytoplasmic lncRNAs act as competing endogenous RNAs (ceRNAs) against miRNAs regulating the release of target mRNAs of miRNAs. In the tumor microenvironment, lncRNAs are aberrantly expressed, breaking the balance of miRNA and target mRNA resulting in the promotion of malignant tumor progression *via* abnormal expression of tumor-promoting or tumor-suppressing genes.

The other way to classify lncRNAs is by the protein-coding gene relative location. First, the *righteous* lncRNAs overlap with exon regions. The *antisense* lncRNAs start with the reverse transcription process of exons. The *bidirectional* lncRNA begins closely with the neighboring protein-coding genes on the antisense strands. The *basal* lncRNA is from intron regions, and the *intergenic* lncRNA resides between different genes on the chromosome (Wang et al., 2021).

LncRNAs can be categorized by their molecular functions, guide, decoy, and backbone molecules. LncRNA is bound to DNA or proteins; decoy molecules inhibit the transcription of downstream genes while guide molecules enhance the transcription. Backbone molecules are scaffold molecules for protein complexes to form nucleic acid-protein complexes involved in epigenetic functions (Han et al., 2022; Nadhan et al., 2022).

**FIGURE 1:**
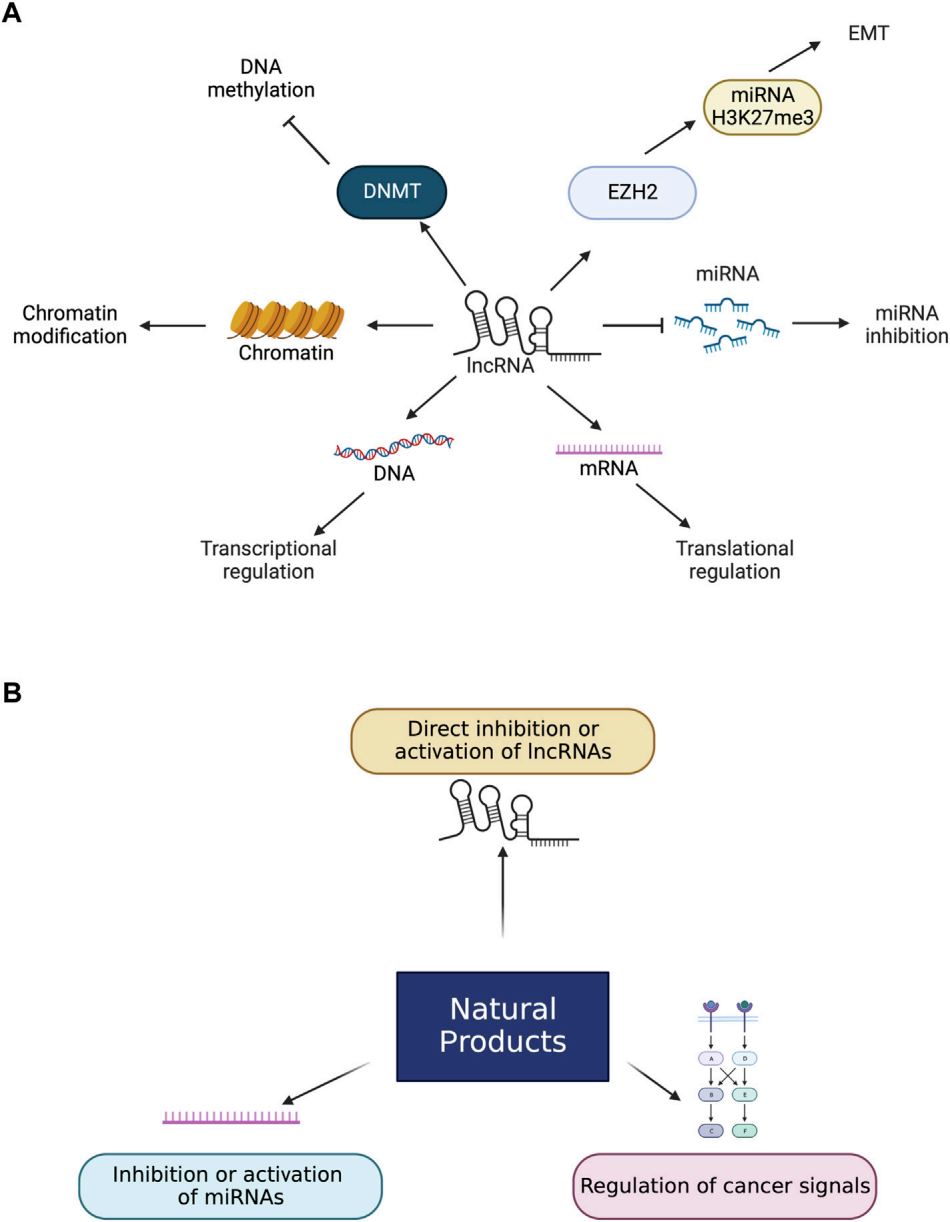
Roles of lncRNAs and natural products in cancers. **(A)** lncRNAs change the level of miRNAs which leads to apoptosis. miRNA inhibition can also lead to buffing the effect of miRNAs. LncRNAs lead to the EMT process by interacting with EZH2 and enhancing H3K27me3 levels. LncRNAs control the transcriptional and translational levels by controlling DNA and mRNA directly and are involved in chromatin modification. LncRNAs bind to DNMT and suppress DNA methylation. **(B)**. Natural products have several biological effects on lncRNAs directly and indirectly. Natural products can directly bind to lncRNAs to enhance or inactivate lncRNAs. Natural products can also control miRNAs and cancer signals that lead to activating or inactivating lncRNAs related to cancers.

The regulatory roles of lncRNAs involve major life events and biological processes like stem cell differentiation, gene expression, development, cell proliferation, and metastasis, so they are closely correlated to the onset and development of cancer and other diseases (Figure 1) (He et al., 2022; Liu et al., 2022; Wang et al., 2022). In recent findings, the detection of lncRNAs is available from patients’ blood to use lncRNAs as biomarkers over tissue lncRNAs. Circulating lncRNAs can be used as potential biomarkers to diagnose several cancers, including CRCs (Badowski et al., 2022).

## 3 LncRNAs and colorectal cancers

### 3.1 Functions of lncRNA in CRCs

In CRC, lncRNA is involved in RNA degradation, splicing, transcription, and translation (Xie et al., 2016). LncRNAs have essential roles in the carcinogenesis of CRCs, such as serving as oncogenes or tumor suppressor genes and interacting with DNAs, RNAs, and proteins (Ragusa et al., 2015). Furthermore, numerous studies revealed that lncRNAs function as endogenous miRNAs to contribute to the competitive endogenous RNA network of tumor regulation (Hashemi et al., 2022; Shen et al., 2022). Control of gene expression in developmental processes and cell differentiation has also been known as a role of lncRNAs (Cao, 2014). Indeed, lncRNAs work as gene expression regulators at epigenetic, transcriptional, and post-transcriptional levels (Xie et al., 2016). Recently, it has been revealed that lncRNAs are involved in the chemoresistance of CRCs through multiple mechanisms, including acting as structural RNAs in scaffolding ribonuclear protein complexes, interacting with other miRNAs, epigenetic modification, and regulating several gene expressions in essential cellular processes such as cell proliferation, differentiation, apoptosis, invasion, and metastasis (Lizarbe et al., 2017). Another interesting point of lncRNAs is acting as competing endogenous RNAs (ceRNAs), inhibiting targets of miRNAs. This leads to regulating miRNAs involved in CRC malignancies, such as migration, invasion, and proliferation (Li et al., 2017).

LncRNAs are commonly found in the serum or plasma of peripheral blood (Xie et al., 2016). Some blood lncRNAs are increased, working as oncogenes in tumor status. Meanwhile, others have tumor suppressor roles (Smolle et al., 2014). Some lncRNAs found in CRC cells and tissues have an increased tendency to correlate with poor prognosis and malignancy in CRC patients (Kam et al., 2014). In recent years, advanced bioinformatics, including microarray and next-generation sequencing, revealed many different lncRNAs are involved in CRC progression (Wang et al., 2015; Arun et al., 2018; Siddiqui et al., 2019). CRC shows the progression similarly to other solid tumors. In stage 0, carcinoma *in situ*, no local lymph node metastasis, and no distant metastasis are observed. In stage I, the tumor invades the submucosa or muscle layer, without local lymph node metastasis or distant metastasis. In stage II, the tumor invades the serosa layer or the large intestine and surrounding tissues through the muscular layer, without peritoneal coverage, local lymph node metastasis or distant metastasis. In stage III, the tumor directly invades other organs, with local lymph node metastasis and no distant metastasis. In stage IV, the tumor directly invades other organs, with local lymph node metastasis and distant metastasis (Fabian et al., 2023). We discuss some of the CRC-related lncRNAs, which can be good candidates to develop new approaches to target CRC, especially using natural products.

### 3.2 LncRNAs related to CRC

More than 80 different lncRNAs have been reported to be directly or indirectly associated with colorectal cancer. The functions and mechanisms are still studied actively to utilize them as prognostic markers or therapeutic targets. Among them, we discuss ten representative lncRNAs, which have been actively studied with obvious evidence affecting both positively and negatively in CRCs in different aspects (Table 1).

**TABLE 1:** lncRNAs associated with colorectal cancers.

lncRNA	Characters	Level	Mechanisms in CRC	References
CASC11	Metastasis, proliferation	↑	c-Myc binding to the promoter region of CAS11 to increase histone of promoter; Interaction with hnRNP-K to activate WNT/β-catenin	Fabian et al. (2023)
CCAT1	Cancer development, invasion, metastasis, carcinogenesis	↓	Superenhancer cMyc transcribes CCAT1	Kam et al. (2014); Ye et al. (2015); Abedini et al. (2019); Xue et al. (2021)
CCAT2	Pathogenesis	↑	Regulation of miR-17-5p, miR-20a, MYC; Modification of WNT signaling	Ma et al. (2015)
CRNDE	Prognostic marker	↑	Correlation with IRX5 mRNA expression	Xu et al. (2014); Liu et al. (2016); Ding et al. (2017)
GAS5	Prognostic marker	↓	GAS5 is regulated by p53	Han et al. (2017)
H19	Prognostic marker	↑	Regulation of CDK8-β-catenin, essential Rb-E2F signaling pathway; Recruitment of eIF4A3; Mediating MTX resistance through WNT/β-catenin signal activation; Modification of EMT; Functioning as a ceRNA for miR138, miR200a	Saus et al. (2016); Schwarzenbach. (2016); Chen et al. (2017)
HOTAIR	Carcinogenesis, prognostic marker	↑	Association with PRC2 function; Modification of EMT	Cui et al. (2002); Meeran et al. (2010); Dou et al. (2016); Luo et al. (2017); Song et al. (2020); Deng et al. (2021)
MALAT1	Metastasis, proliferation	↑	Interaction with CC chemokine ligand 5; Promotion of SFSF1 phosphorylation to enhance AKAP-9	Yang et al. (2015); Schmitt and Chang (2016)
PCAT-1	Prognostic marker, proliferation	↑	Promotion of PRC2	Ge et al. (2013); Zhao et al. (2015); Zhao et al. (2016)
UCA1	Carcinogenesis; chemoresistance; prognostic marker	↑	Inhibition of miR-204-5p; Regulation of glucose metabolism	Han et al. (2014); Bian et al. (2016); Qiao et al. (2017)

#### 3.2.1 Cancer susceptibility candidate 11 (CASC11)

CASC11 is located on chromosome 8q24. CASC11 lncRNA has been reported to be increased in CRC cells and tissues. Furthermore, the tumor size correlates with the expression level of CASC11 (Zhang et al., 2016). When CASC11 is inhibited in CRC, proliferation and metastasis are suppressed in tumor cells by interacting with heterogeneous ribonucleoprotein. This leads to the protection of β-catenin degradation and increases the transcription activity (Shen et al., 2017). CASC11 has been reported to suppress Wnt signaling in colorectal cancer (Javed et al., 2020).

#### 3.2.2 Colon cancer-associated transcript 1 (CCAT1)

CCAT1 has been recently identified as a lncRNA correlated with colorectal adenomas and adenocarcinomas at any stage (Ye et al., 2015). CCAT1 is a good target for real-time *in vivo* imaging techniques (Kam et al., 2014). CCAT1 has an oncogenic role in activating Myc, the target of miR-155, and promotes cell proliferation and invasion through direct interaction with the promoter region (Wang et al., 2015). A study reported that CCAT1 is increased in gallbladder cancer tissues, and this is through knocking-down miRNA that is related to tumor cell invasion and proliferation (Ma et al., 2015).

#### 3.2.3 CCAT2

CCAT2 interacts with TCF7L2 and leads to the enhanced expression of Myc. Then, Myc regulates miR-17p and miR-20a functions. The output of this process is genomic instability and the promotion of cancer malignancy (Wu et al., 2016). Since CCAT2 has shown the modification of clinical outcomes, CCAT2 is considered an excellent target for lncRNA therapies and a diagnostic marker of CRC (Catana et al., 2017). Moreover, CCAT2 is critical in the loop formation between genomic DNA locus rs6983267 and Myc promoter, which turns on the oncogenic activity of Myc (Xu et al., 2014).

#### 3.2.4 Colorectal neoplasia differentially expressed (CRNDE)

CRNDE is differently upregulated in CRC tissues in 90%. Insulin and IGFs induce the Warburg effect in cancer cells by metabolic changes that regulate CRNDE (Ye et al., 2015). In a recent finding, CRNDE-h (transcript variant one of CRNDE) was highly upregulated in CRC tissues. The overexpression levels were positively correlated with the degree of malignancy, such as tumor size, lymph node metastasis, distant metastasis, and survival rate (Liu et al., 2016; Ding et al., 2017). The knockdown study of CRNDE showed the apoptosis of CRC cells *in vitro* and *in vivo* (Ding et al., 2017). Furthermore, the knockdown of CRNDE with miR-181a-5p showed the inhibition of cell proliferation and the reduction of chemoresistance *via* the downregulation of Wnt/β-catenin signaling (Han et al., 2017).

#### 3.2.5 Growth arrest-specific transcript 5 (GAS5)

GAS5 is a lncRNA with tumor suppressor function. GAS5 interacts with the intracellular glucocorticoid receptor and regulates cellular metabolism and survival (Kino et al., 2010). Recent findings revealed that GAS5 is suppressed in several different cancers, and the downregulation of GAS5 was accompanied by the advanced TNM stage and large tumor size in CRC (Saus et al., 2016).

#### 3.2.6 H19

H19 is a lncRNA enhanced in the early stages of embryogenesis and suppressed after birth (Ariel et al., 1998). H19 was first identified from the transcript of a gene cluster, H19/insulin-like growth factor 2 (IGF2), more from the maternal allele than the paternal (Chen et al., 2017). H19 regulates several cancer-associated proteins, including ubiquitin E3 ligase family, a retinoblastoma tumor suppressor, and calneuron 1 (Schwarzenbach, 2016). Furthermore, the methylated region of H19 and the upstream of IGF2 exon three were hypomethylated (Cui et al., 2002). The upregulation of H19 is correlated with the high TNM stage and poor prognosis (Chen et al., 2017).

#### 3.2.7 HOX transcript antisense intergenic RNA (HOTAIR)

HOTAIR binds with polycomb repressive complex 2 (PRC2) in trans and changes cellular gene expression and epigenetics (Dou et al., 2016; Xie et al., 2016). HOTAIR is upregulated in epithelial cancer cells, inducing histone methylation and cancer cell invasion (Svoboda et al., 2014). Furthermore, HOTAIR upregulation is closely related to the proteins associated with the malignancy of CRCs, such as angiogenesis, invasion, metastasis, and high tumor stage, i.e., E-cadherin, vimentin, and matrix metalloproteinase (Luo et al., 2017). The correlation of HOTAIR with cancer malignancy and poor prognosis is related to CRC and several cancers like pancreatic cancer, epithelial ovarian cancer, mammary cancer, and hepatocellular carcinoma (Deng et al., 2017). A study showed the correlation between HOTAIR and poor prognosis using CRC blood, and tissue samples suggested this lncRNA as a prognostic marker for sporadic CRC (Svoboda et al., 2014).

#### 3.2.8 Metastasis-associated lung adenocarcinoma transcript 1 (MALAT1)

MALAT1 regulates alternative splicing through pre-mRNA binding to localize transcriptionally active genes in chromatin with serine/arginine splicing factor (Schmitt and Chang, 2016). MALAT1 activates AKAP-9, which leads to the malignancy of several cancers, such as melanoma, breast cancer, thyroid cancer, oral cancer, lung cancer, and colorectal cancer, through enhanced cell proliferation, migration, invasion, and metastasis (Yang et al., 2015). In CRC cells, MALAT1 promotes SPRK1 expression and SRSF1 phosphorylation, which leads to the upregulation of AKAP-9 expression (Hu et al., 2016).

#### 3.2.9 Prostate cancer-associated ncRNA transcript 1 (PCAT-1)

As the terminology, PCAT-1 was first identified in prostate cancer, but this lncRNA has also been reported to be related to CRCs’ metastasis (Zhao et al., 2016). PCAT-1 promotes the expression of PRC2, which induces cell proliferation in cancer cells *in vitro* (Smolle et al., 2014). Additionally, PCAT-1 is involved in non-small cell lung cancer to upregulate cancer cell proliferation, invasion, and migration (Zhao et al., 2015). In CRC, PCAT-1 expression is highly correlated with distant metastasis, patient survival, and prognosis (Ge et al., 2013). In a recent study, PCAT upregulation in CRC enhanced c-myc signaling. At the same time, CRC deficiency decreased proliferation and blockage of the cell cycle *via* the suppression of c-myc and cyclins (Qiao et al., 2017).

#### 3.2.10 Urothelial carcinoma-associated 1 (UCA1)

UCA1 is a lncRNA with the character of oncofetal genes that are involved in embryonic development (Han et al., 2014). However, bladder cancer is where UCA1 is highly expressed; UCA1 has been reported to be upregulated in CRC cells to inhibit apoptosis and develop tumorigenesis (Bian et al., 2016). UCA1 has a critical role in cancer biologies, such as cell transformation, proliferation, invasion, mortality, and chemoresistance (Wang et al., 2008). Furthermore, the UCA1 expression level is correlated with the tumor size. Meanwhile, CRC tumor size is reduced when UCA1 is deficient (Han et al., 2014).

## 4 Targeting lncRNAs with natural products for potential CRC treatment

Natural products and their derivatives have been widely studied and applied as anticancer agents for several decades (da Rocha et al., 2001). Natural products and their derivatives have various potent biological activities such as anticancer, anti-inflammatory, pro-apoptotic, and antioxidant characteristics, with the potential for chemotherapies and chemo-preventions for several cancers. They show anticancer effects primarily through epigenetic change, regulation of signaling pathways, and miRNA regulation in cancer cells or tissues (Homayoonfal et al., 2021). Below we introduce several practical natural products employed in cancer treatment targeting lncRNAs introduced previously (Table 2).

**TABLE 2:** Regulation of lncRNA by natural products in CRC.

Natural compound	Target lncRNA	Effect	Targeting mechanisms	References
Berberine	HOTAIR	↓	Inhibition of EMT	Song et al. (2020); Zhong et al. (2022)
Calycosin	HOTAIR	↓	Induction of apoptosis	Wu et al. (2019); Deng et al. (2021)
Curcumin	H19	↓	Inhibition of EMT, transcriptional regulation	Ashrafizadeh et al. (2020)
	GAS5	↑	Transcriptional regulation	Grynkiewicz and Slifirski, (2012)
	HOTAIR	↓	Inhibition of migration	Ashrafizadeh et al. (2020)
DIM	HOTAIR	↓	Inhibition of autophagy	Zhang et al. (2014)
Gambogic acid	GAS5	↑	Transcriptional regulation	Lee et al. (2015); Che Hassan et al. (2018); Gao et al. (2021)
Genistein	HOTAIR	↓	Transcriptional regulation, chromatin remodeling	Meeran et al. (2010); Ravishankar et al. (2013)
Ginsenoside	HOTAIR	↓	Inhibition of proliferation and invasion	Abedini et al. (2019); Xue et al. (2021)
	H19	↓	Inhibition of proliferation and invasion	Li and Qi, (2019)
Quercetin	MALAT1	↓	Transcriptional regulation	Reyes-Farias and Carrasco-Pozo (2019); Zhang et al. (2019)
Resveratrol	HOTAIR	↓	Transcriptional regulation, chromatin remodeling	Cimino et al. (2012)
	MALAT1	↓	Induction of apoptosis	Vallino et al. (2020)
	GAS5	↓	Inhibition of proliferation and invasion	Cimino et al. (2012)
	UCA1	↓	Transcriptional regulation	Cimino et al. (2012)

### 4.1 Berberine

Berberine is a pentacyclic isoquinoline alkaloid compound isolated from *Berberis* genus plants. The broad pharmacological application of berberine includes anticancer, antidiabetic, anti-obesity, and cardioprotective effects (Zhong et al., 2022). This compound interacts with specific receptors, ligands, and biological enzymes leading to anti-inflammatory and antioxidant activities (Song et al., 2020). Like other natural products, berberine modulates lncRNAs to inhibit cancer progression. In a recent study, the combination treatment of berberine with gefitinib downregulated HOTAIR function to enhance miR-34a-5p. The upregulation of miR-34a-5pupregulatess E-cadherin, to the arrest of EMT, invasion, and migration by SNAIL-mediated E-cadherin increase in lung cancer cells (Zheng et al., 2020). As the effect of miR-34a-5p is involved in colorectal cancer, berberine can be a supportive candidate to treat colorectal cancer.

### 4.2 Calycosin

Calycosin (C_16_H_12_O_5_) is an isoflavone phytoestrogen isolated from the dried roots of Radix astragali with several biological effects (Wu et al., 2019). The anticancer effect of calycosin has been vigorously studied in several different cancers, such as breast cancer, liver cancer, colorectal cancer, and osteosarcoma (Deng et al., 2021). In breast cancer, calycosin downregulated phosphorylation of Akt and its downstream lncRNA, HOTAIR. This effect strongly decreased cancer development (Chen et al., 2015). This result was from breast cancer cell line MCF-7 downregulating EGFR and ERK1/2 with suppressed proliferation and enhanced apoptosis. Although MCF-7 is not a colorectal cancer cell line, the onset of colorectal cancer shares the effect of the EGFR signaling pathway, and calycosin can be an excellent synergetic candidate for current therapies.

### 4.3 Curcumin

The chemical formulation of curcumin (diferuloylmethane) is C_21_H_20_O_6_. This is a polyphenol compound with bright yellow color isolated from Curcuma longa (the rhizome of turmeric) (Ashrafizadeh et al., 2020). This compound has been utilized as a traditional herbal medicine in Eastern society for a long time. The unique structure of curcumin enables suppression of ROS generation and several different pharmacological properties such as anticancer, neuroprotective, cardioprotective, hepatoprotective, anti-analgesic, and anti-inflammatory effects. Since it has been reported that curcumin targets lncRNAs, many groups have focused on this compound and are being actively studied (Grynkiewicz and Slifirski, 2012). In a recent study, As mentioned previously, the curcumin-pretreated cancer cells showed the activation of GAS5 promotors, while GAS5 is downregulated in CRC (Zheng et al., 2021). Further studies regarding the effect of curcumin targeting GAS5 have not been studied rigorously, but this can be an extraordinary therapeutic approach when more preclinical and clinical studies are fulfilled.

### 4.4 3,3′-diinodolymethane (DIM)

DIM (C_17_H_14_N_2_) is a phytochemical in several cruciferous vegetables like cabbage, broccoli, lettuce, and kale (Licznerska and Baer-Dubowska, 2016). DIM modulates various signaling pathways to induce proliferation, cell survival, apoptosis, and angiogenesis (Zhang et al., 2014). DIM, directly and indirectly, downregulates Akt/FOXM1 signaling pathway and suppresses cancer progression and metastasis (Cai et al., 2015). The downregulation of Akt/FOXM1 leads to the decreased expression of lncRNAs, such as HOTAIR and CCAT1-L, that are highly involved in colorectal cancers, and cancer malignancy has been regulated through this pathway (Zinovieva et al., 2017). More vigorous studies about DIM are required to develop CRCs, but this is a very hopeful candidate for future therapeutics.

### 4.5 Gambogic acid (GA)

GA is a brownish resin and the most potent compound of gambose, isolated from Garcinia hanburyi (Che Hassan et al., 2018). GA has been used as a traditional medicine with various biological activities such as anticancer, anti-inflammatory, and antiviral effects with extremely minimal toxicity (Lee et al., 2015; Gao et al., 2021; Xu et al., 2022). When cancer cells are treated with GA, GAS5 expression is increased, which leads to the downregulation of EZH2 by binding E2F4. The downregulation of EZH2 enhances miR-101. miR-101 has a pro-apoptotic property that consequently suppresses cancer cell invasion and progression in preclinical stages.

### 4.6 Genistein

Genistein (C_15_H_10_O_5_) is a phytoestrogen-originated isoflavone derived from soy. Phytoestrogens are non-steroidal herbal components with structures like estrogen functioning estrogen-like or anti-estrogenically (Ravishankar et al., 2013). The biological activities of genistein include tyrosine kinase inhibition, anticancer, and antioxidants. The anticancer function of genistein affects various cellular processes such as angiogenesis, apoptosis, and cell cycle (Meeran et al., 2010). One of the targets of genistein to have an anticancer effect is epigenetic changes affecting cancer-associated genes, including lncRNAs (Imai-Sumida et al., 2020). Genistein downregulates EED levels in PRC2, followed by the inhibition of the interaction between HOTAIR and PRC2. The suppression of HOTAIR/PRC2 recruitment to the promoter region of ZO-1 leads to the increased transcription of ZO-1. The other effect of genistein is the inhibition of SNAIL transcription by suppressing the interaction between HOTAIR and SMARCB1. The reduced HOTAIR interaction with chromatin remodeling factors leads to the repression of HOTAIR/chromatin remodeling pathways, followed by the downregulation of cancer malignancy (Imai-Sumida et al., 2020).

### 4.7 Ginsenoside

Ginsenoside is one of the steroid glycoside fractions, triterpene saponin from ginseng roots (Nakhjavani et al., 2019). Ginsenoside is not a single compound, but more than ten molecules have been identified. According to the number of hydroxyl groups in its chemical structure, ginsenoside is subcategorized into two major classes. The first group is protopanaxatriol (PPT), with six positions occupied by hydroxyl groups, and the next group is protopanaxadiol (PPD), with six positions not occupied by hydroxyl groups. The members of PPT are Re, G1, Rg2, and Rh1 and of PPD are Rb1, Rb2, Rb3, Rc, Rd, Rg3, and Rh2. Ginsenoside molecules have various biological activities showing slight differences between each molecule (Xue et al., 2021). It has been studied that CCAT1 is highly expressed in several CRCs affecting cell proliferation, invasion, and migration (Abedini et al., 2019). Especially CCAT1 suppression by ginsenoside-Rg3 reduced the signaling of the PI3K/Akt pathway, followed by suppression of CRC development (Li and Qi, 2019). In addition, ginsenoside Rg3 suppressed cancer development by suppressing HOTAIR in hepatoma (Pu et al., 2021).

### 4.8 Quercetin

Quercetin (3,5,7,30,40-pentahydroxyflavone) is a natural flavanol ubiquitously found in fruits and vegetables, which can be found as one of the easiest in the western diet (Hertog and Hollman, 1996; Boots et al., 2008). Various biological activities of quercetin include anticancer, antidiabetic, anti-inflammatory, and antioxidant effects (Carullo et al., 2017). Arresting cell cycle, anti-proliferation, and apoptosis functions are remarkable anticancer effects of quercetin. Furthermore, it has been reported that various lncRNAs are affected by quercetin in cancer cells and tissues (Reyes-Farias and Carrasco-Pozo, 2019). A study reported quercetin could alter the expression of 240 lncRNAs along with 1,415 mRNAs, 83 miRNAs, and 131 circRNAs through the analysis of HCT-116 colorectal cancer cell line with MTS assay and flow cytometry (Zhang et al., 2019). Additionally, quercetin suppresses the expression of MALAT1 and MIAT, followed by decreased cell survival (Esteghlal et al., 2021). So, in experimental studies, quercetin may be applied to CRCs to inactivate PI3K/Akt signaling pathway by reducing the expression of lncRNAs, MALAT1, and MIAT.

### 4.9 Resveratrol

Resveratrol (C_14_H_12_O_3_, trans-3,5,4′-trihydroxystilbene) is a natural polyphenolic phytoalexin isolated from various foods, including red wine, berries, grapes, nuts, and else (Bishayee, 2009). Resveratrol shows anticancer effects targeting multiple signaling molecules leading to the suppression of cancer cell viability and growth with minimal toxicity (Cimino et al., 2012). Resveratrol increases tumor suppressive lncRNAs such as GAS5, HULC, UCA1, and PVT1 in several cancers (Vallino et al., 2020). Furthermore, resveratrol decreased MALAT1 expression, followed by the Wnt/β-catenin signaling pathway reducing tumor progression in CRC, showing a reduced transformation, invasion, and metastasis, and further studies may lead to the development of a new therapeutic candidate (Ji et al., 2013).

## 5 Conclusion and future perspectives

Discovery and studying novel therapeutic reagents are extremely difficult and time-consuming. Many methodologies and strategies have been applied in cancer biology for a long time, which will continue forever as long as humans exist on earth. These difficulties are from various cancer types, locations, oncogenic mechanisms, and others. People have already developed various effective chemo-reagents to treat and prevent cancers, but most are partially effective. Furthermore, the mediocre effect and inappropriate potential of chemotherapies could be the reason for cancer recurrence. The paradigm shift from sole chemotherapy to chemoprevention with chemotherapy was first elected in breast cancer. Further desperate trials of chemoprevention and chemotherapies have been made to understand detailed signaling molecules and pathways as the target of effective natural products.

LncRNAs are one of the non-protein coding RNA classes that affect several cancer-related cellular processes such as proliferation, differentiation, and apoptosis. After many lncRNAs have been known as tumor suppressive or oncogenic, many groups started focusing on small molecules such as phytochemicals and natural compounds to treat cancer targeting those lncRNAs. We discussed several lncRNAs related to CRC and possible natural products to regulate those lncRNAs. The biggest hurdles to applying these natural compounds for chemotherapy are experiments’ challenging time and effort to confirm their activity and clinical challenges. Neither laboratory experiment nor clinical exam is enough to develop a new chemotherapeutic natural compound. Proper animal experiments should follow up for *in vivo* analysis to prove and support the preliminary *in vitro* data for establishment. Many of the lncRNAs and natural products discussed in this review have not been clearly studied in CRC; however, considering the many sharing mechanisms of CRC with other cancers, the lncRNAs and natural products have a high chance of being one of the critical factors in onset and progression in CRCs.

Additionally, low bioactivity, short availability, poor solubility, and a delivery method must be considered not to restrict the efficacy of natural products in clinical studies and *in vivo* experiments. Successful collaborative studies by nanotechnologists, chemists, biologists, and physicians will promise to overcome the hurdles to developing natural products for applicable chemotherapeutics. Another recommended method to optimize the stated approaches is electing the combination method. Various mixture therapies have been studied and tried in current cancer biology to treat cancer, and the combination of various natural products or with other anticancer agents as well as adjuvants with proved to have low toxicity. Since most of the studies were carried out experimentally and they show discrepancies in results by groups, it is important to study more about lncRNAs and natural products to standardize as a diagnostic marker and therapeutic purpose.

The concept of transitioning chemotherapy to chemoprevention has been first suggested in treating breast cancer to prevent recurrent cancers. Afterward, several studies have been made for a while to detect proper signaling molecules as a target of functional natural compounds. More detailed biochemical studies should be conducted to reveal the correct mechanisms to prevent unwanted actions of natural products in the future.

Natural products are highly available, inexpensive, and low toxic, with minimal side effects. These phytochemicals can be regarded as an innovative and promising field for developing new therapeutic strategies to overcome colorectal cancer and other cancers with minimal recurrence after treatment.
